# Quick-EXAFS implementation on the general purpose EXAFS beamline at ESRF

**DOI:** 10.1107/S0909049510046546

**Published:** 2011-01-18

**Authors:** Carmelo Prestipino, Olivier Mathon, Ricardo Hino, Antonia Beteva, Sakura Pascarelli

**Affiliations:** a European Synchrotron Radiation Facilities, Grenoble, France; b CNRS UMR 6226, France

**Keywords:** time-resolved XAFS, heterogeneous catalysis, powders, chemical processes

## Abstract

The new implementation of QEXAFS acquisition on the general purpose EXAFS beamline BM29 at the European Synchrotron Radiation Facility is presented. By adopting a continuous-scan mode, a high signal-to-noise ratio can be maintained, together with a fixed exit beam and full compatibility with step-by-step operation, while reducing acquisition times to a few seconds. The new equipment implemented on the beamline is described and the potential of this application is demonstrated with a few examples.

## Abstract 2

The new implementation of QEXAFS acquisition on the general purpose EXAFS beamline BM29 at the European Synchrotron Radiation Facility is presented.

## 1. Introduction

Time-dependent studies are of particular interest in solid-state chemistry and heterogeneous catalysis as they provide information on kinetics and on the mechanisms of chemical reactions and phase transitions. Many techniques have been successfully applied for time-resolved studies. Among them, EXAFS spectroscopy plays an important role because of its inherent advantages for *in situ* characterization, since hard X-rays can penetrate highly absorbing sample environments (liquid cells, high-pressure and/or high-temperature cells, chemical reactors, *etc.*). Moreover, EXAFS is element specific and probes the local structure in different states of matter (solid, amorphous, liquid, gas) with the same degree of accuracy (Teo, 1981 ▶, 1986 ▶; Koningsberger & Prins, 1987 ▶).

The BM29 spectrometer at ESRF is a general purpose EXAFS beamline that has shown its reliability in several domains of science (Egry *et al.*, 2008 ▶; Poloni *et al.*, 2008 ▶; Dent *et al.*, 2008 ▶). Unfortunately the time necessary to collect a full conventional (step-by-step) EXAFS spectrum spans from 20 min to 1 h, limiting the domains of application of the beamline to time-resolved studies with slow kinetics (Le Toquin *et al.*, 2006 ▶). In order to study faster evolving systems, it is fundamental to implement faster techniques.

During the last 30 years, in order to reduce the time necessary to collect a full EXAFS spectrum, several experimental approaches have been developed. Two of the more successful ones are energy-dispersive geometry (EDXAS) (Matsushita & Phizackerley, 1981 ▶; Flank *et al.*, 1982 ▶) and quick-EXAFS acquisition (Frahm, 1989 ▶; Als-Nielsen *et al.*, 1995 ▶).

The energy-dispersive method allows all data points of an EXAFS spectrum to be recorded in parallel, and the time resolution is limited only by the detector readout time. Much work has been carried out by different groups (Douillard *et al.*, 1996 ▶; Lamberti *et al.*, 2002 ▶; Suzuki *et al.*, 2003 ▶; Dent *et al.*, 2007 ▶; Newton *et al.*, 2007*a* ▶,*b* ▶). However, energy-dispersive EXAFS presents some intrinsic limitations (Pascarelli *et al.*, 1999*a* ▶): (i) the energy range of the spectra is severely restricted at low energies, (ii) samples need a microscopic homogeneity (Dent, 2002 ▶) and must not destroy the energy–direction correlation of the polychromatic incident beam, and (iii) the technique is restricted to work in transmission and does not allow EXAFS to be measured using detection of de-excitation channels (total electron yield or fluorescence). The last two limitations can be overcome at the expense of a compromise between photon flux and energy by the use of the TurboXAS acquisition mode (Pascarelli *et al.*, 1999*b* ▶, 2000 ▶; Nagai *et al.*, 2008 ▶).

The alternative method, the quick-EXAFS (or QEXAFS) method, consists of a continuous scan of the angle of the crystal monochromator during acquisition. The method evolved in parallel to energy-dispersive technology with particular care for the multi-technique approach by several groups in different laboratories (Clausen, 1998 ▶; Als-Nielsen *et al.*, 1995 ▶; Hecht *et al.*, 1996 ▶; Grunwaldt *et al.*, 2001 ▶, 2009 ▶; Rickers *et al.*, 2007 ▶; Stotzel *et al.*, 2008 ▶; Bauer *et al.*, 2010 ▶). This configuration, although immune to the limitations encountered with the energy-dispersive geometry, was limited to a time resolution of a few seconds per spectrum. Recently this acquisition method evolved in two directions to achieve a time resolution lower than 1 s: (i) piezo-QEXAFS (Richwin *et al.*, 2001 ▶), able to collect an XANES spectrum in less than 10 ms and an energy range that could reach almost 1000 eV, and (ii) cam-driven QEXAFS (Frahm *et al.*, 2005 ▶), with no more constrain on energy range, and a time resolution that could reach 12.5 ms per scan but with a longer energy range. The increase in time resolution for Q-EXAFS has been accompanied by new disadvantages such as the loss of the fixed exit and a limited choice of energy range, which is dictated by the range of the piezoelectric transducer or the available eccentrics. In fact, to reach this time resolution, a channel-cut monochromator is utilized, moved by a cam-driven eccentric or a piezoelectric motor (Richwin *et al.*, 2001 ▶; Frahm *et al.*, 2005 ▶).

In this paper we present the implementation of QEXAFS on BM29, the general purpose EXAFS beamline of the European Synchrotron Radiation Facility (ESRF). With this development we wish to provide the user community with a complementary capacity that allows the acquisition time to be reduced drastically (roughly from minutes to seconds) with a signal-to-noise ratio comparable to the step-by-step mode, without losing the fixed exit option, and maintaining full compatibility with the step-by-step acquisition. Although not sufficient to study fundamental mechanisms of chemistry on the nanosecond to picosecond timescales, which can now be tackled using a pump and probe approach (Oyanagi *et al.*, 2001 ▶; Chen, 2001 ▶; Seres & Spielmann, 2008 ▶), the time resolution of seconds presents many advantages: (i) the number of photons transmitted by materials with ordinary X-ray absorption coefficients is sufficiently large to achieve reasonable statistics, (ii) the response time of common ionization chambers is sufficient (for a faster response time the ionization chambers should be *ad hoc* optimized), (iii) many solid-state chemistry transformations happen on this timescale. In fact, time-resolved EXAFS on timescales of a few seconds to minutes has been applied successfully to investigate many systems, such as polymerization (Epple *et al.*, 1996 ▶, 1997 ▶), precipitation (Hilbrandt & Martin, 1997 ▶) and crystallization processes (Dacapito *et al.*, 1993 ▶). Our facility would be very complementary to other faster time-resolved EXAFS beamlines and would allow an interesting portion of scientific applications to be covered.

The motivation behind the implementation of the continuous-scan mode for EXAFS came from the realisation that a large fraction of the time needed for a conventional step-by-step EXAFS scan is spent waiting for the monochromator mechanics to move, for mechanical vibrations to settle, for thermal stabilization and for the readout time of different devices, such as motor encoders and temperature or pressure sensors (Richwin *et al.*, 2001 ▶), and often the time devoted to photon detection is only a negligible portion of the total.

## 2. Hardware and software solution

The BM29 spectrometer is installed on an ESRF bending magnet with a critical energy of 20.6 keV. The optical layout is composed of a KOHZU double-cam fixed-exit monochromator followed by a mirror for vertical focusing and harmonic rejection. During a QEXAFS scan the monochromator position is recorded by measuring the number of steps sent to its stepper motor. This procedure has proven to be equivalent to the reading of the angular encoder tested during step-by-step acquisition. The only exception is when QEXAFS scans are collected both up and down stream at the same energy range. In such case the raw spectra in one direction are shifted compared with the other, owing to the mechanical backlash. However, it is a constant number of steps, which does not depend on the energy range and is automatically corrected by the acquisition software. Typical detectors used for transmission experiments are the three ionization chambers with remarkable accuracy conceived *ad hoc* by Pettifer *et al.* (1999 ▶). These ionization chambers have proved to be able to operate with noise levels close to the theoretical photon-counting limit (Pettifer *et al.*, 1999 ▶) and with a response time sufficient for acquisition on a timescale of seconds. The number of photons arriving at the sample for a standard beam size (5 mm width) is in the 10^10^ photon s^−1^ range. In order to maintain a high signal-to-noise ratio (equivalent to that of the step-by-step scan) while continuously scanning the crystals, the following requirements are mandatory: (i) the beam position must remain fixed during the scan and (ii) the acquisition electronics must be fast and accurate.

### 2.1. Stabilization of beam position

The first condition mainly affects the noise level. If the sample homogeneity or thickness is not perfect, a movement of the beam on the sample will lead to changes in the amount of matter probed. This effect is well known and sample homogeneity is a crucial parameter for high-quality EXAFS measurements (Stern & Kim, 1981 ▶; Lu & Stern, 1983 ▶; Babanov *et al.*, 2001 ▶). However, it is not always possible to employ homogeneous samples. Many samples cannot be ground or mixed with an inert matrix and during *in situ* experiments materials can undergo modifications in shape or dimension. In practice, if the sample cannot be optimized the only possibility of obtaining reliable data is to limit as much as possible the movement of the beam during the scan.

The first requirement of obtaining a fixed exit beam is to adopt a suitable optical set-up. BM29 is already equipped with a KOHZU double-cam fixed-exit monochromator (Filipponi *et al.*, 2000 ▶). This monochromator has been demonstrated to have a remarkable beam stability (De Panfilis *et al.*, 2002 ▶), with a displacement of 0.05 mrad over the whole angular range (40°), corresponding to a maximum displacement of 12.5 µm per degree of the X-ray beam on the sample. Displacement could be further reduced (if necessary) by using the super cam option available on the BM29 monochromator. For this kind of optics the most important aspect for a fixed exit is the control of the angle between the two monochromator crystals (Δθ_*d*_). In fact the vertical displacement of the beam (Δ*h*) is a function of this angle and of the sample–monochromator distance (*d*_sample_) as described by (1),
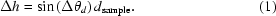
On BM29 during a step-by-step scan the angle θ_*d*_ is controlled by a logical feedback (Filipponi *et al.*, 2000 ▶) that drives a piezoelectric transducer (PZT) link to the first crystal. This feedback monitors the beam intensity at each energy point and corrects θ_*d*_ to match the calculated intensity. The slow reaction time makes this feedback unsuitable for fast continuous scans; therefore a dynamical feedback is mandatory to fine control θ_*d*_ during QEXAFS acquisitions. A remarkable example of dynamical feedback was developed on BM30 (ESRF) (Proux *et al.*, 2006 ▶) based on lock-in detection (Cova *et al.*, 1979 ▶). It presents two interesting features: (i) it works on the top of the rocking curve, *i.e.* with crystals fully parallel; (ii) it is independent of the absolute value of the beam intensity (*I*_0_). This feature allows the feedback to work properly also in the presence of monochromator glitches and during refilling. With respect to the BM30 feedback, on BM29 we have opted for a fully integrated system with implemented *ad hoc* internal software, developed at the ESRF, the MOCO2 controller, which allows a faster response and an easier calibration of controlling parameters. The main idea driving the development of this kind of feedback is to obtain a signal that is proportional to the derivative of the monochromator rocking curve and then correct Δθ_*d*_ to maximize the beam intensity by minimizing the derivative.

In more detail, the intensity of the beam is modulated by applying an additional sinusoidal voltage varying with time (*t*), of amplitude *V*_*a*_ and frequency ω, to the PZT controlling θ_*d*_ [equation (2)],
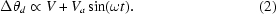
The beam intensity is then modulated at a frequency equal to the exciting frequency ω and amplitude proportional to the first derivative of the rocking curve with respect to the PZT voltage *f*′(V) [equations (3) and (4)],
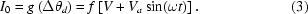
If *V*_*a*_ is small, by the Taylor expansion,
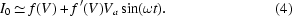
The *I*_0_ detected signal will be finally multiplied by a digital square wavefunction *S*(ω*t* + ϕ) of intensity π/4 with the same frequency (ω) and phase (ϕ) of the sinusoidal voltage used to modulate *I*_0_, and numerically integrated for a time *T* much larger than the oscillation period [equation (5)],
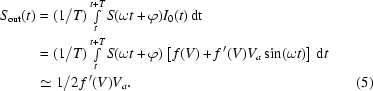
For the properties of orthogonality of sinusoidal functions, the resulting signal *S*_out_ will be close to 1/2*f*′(*V*)*V*_*a*_ [the derivation of equation (5) is shown in the supplementary information^1^]. Consequently *S*_out_ is a function of the derivative of the rocking curve *f*′(*V*), and *S*_out_ = 0 when *f*′(*V*) = 0, *i.e.* when the crystals are parallel. The more robust algorithm for keeping the value of *S*_out_ equal to zero acting on the voltage of the PZT (Δ*V*) is the integral control algorithm [equation (6)], where *f*′′(*V*_0_) is the value of the second derivative at the maximum of the rocking curve and τ is a term that smooths the correction term,
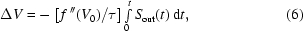

               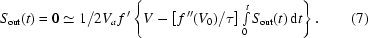
One advantage of our method derives from the fact that the three steps of the feedback, *i.e.* (i) modulation of PZT; (ii) lock-in detection, (iii) minimization of *f*′(*V*), are all performed by the same control card (the MOCO2) allowing a much easier tuning of the parameters.

### 2.2. Acquisition electronics

Fast and reliable acquisition electronics are required to decorrelate the spectrum acquisition time and energy resolution. In fact, the monochromator rotation speed (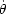) is limited by the minimum acquisition time (Δ*t*), which is related to the energy sampling (Δ*E*). This quantity needs to be maintained comparable or smaller than the intrinsic resolution of the absorption process (Δ*E*_core_), as usually requested for XANES measurements.

**Figure 1:**
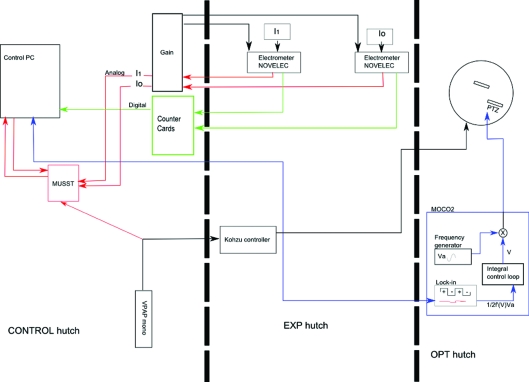
New electronics and acquisition chain implemented on BM29. Red lines represent the new link for performing QEXAFS, the blue lines represent the connection for the feedback, and the green lines represent the old acquisition method for standard step-by-step acquisition

For a chosen Δ*E*, the smaller the value of Δ*t* the faster a spectrum can be collected, as shown in equation (8) where *d* is the *d*-spacing of the monochromator crystal reflection, 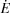 is the speed of the energy scan and θ is the monochromator angle,
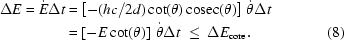
Neglecting photon statistics considerations, the minimum acquisition time is clearly dependent on the kind of electronics used to record the detector signals. On BM29, during a transmission step-by-step scan, as in other facilities, the output signals of the three ionization chambers are amplified and converted from voltage to frequency (VTF) by NOVELEC electrometers and transmitted to the counters cards (Filipponi *et al.*, 2000 ▶), as shown in Fig. 1 ▶ (green line). This acquisition method has shown a remarkable linearity between the signal obtained by the detector and the integration time, but to obtain reliable results it needs a minimum integration time dependent on the range of frequency used by the VTF module. As already pointed by the group of Frahm (Bornebusch *et al.*, 1999 ▶), present electronics using VTF converters do not allow minimum Δ*t* values compatible with acquisitions within the seconds timescale to be obtained. The solution is to convert the signal from analog to digital after amplification.

In our BM29 QEXAFS implementation we use the second output of the NOVELEC electrometer that delivers an analog amplified signal (a voltage) proportional to the detector output current. This analog signal is converted to digital and recorded with the corresponding position of the monochromator by the MUSST module developed at ESRF (Hino *et al.*, 2011 ▶). This scheme is depicted in Fig. 1 ▶ (red line). The MUSST card is a NIM module intelligent controller with its own programming language that produces triggered patterns synchronized with external events with built-in data storage capability. Using this card we are able to simultaneously collect an array of detector signals and motor positions. The MUSST has a very short acquisition time (∼2.5 × 10^−6^ s), which allows a minimum energy sampling Δ*E* [see equation (8)] of the order of magnitude of a hundredth of an electronvolt to be achieved for the fastest available BM29 monochromator rotation speed (0.42° s^−1^).

The electronics chain is represented in Fig. 1 ▶. Note that the signals from the detectors, after the amplification by the NOVELECs, follow two separate chains (red line or green line) allowing switching from a ‘step-by-step mode’ to a ‘continuous-scan mode’ freely without changing any electronics module.

Finally we have developed a software, based on SPEC (Certified Scientific Software), to run the acquisition and retrieve the data. The software loads the acquisition program into the MUSST card and specifies (i) the signals to record, (ii) the sampling frequency (Δ*t*) and (iii) the start and end of acquisition. In parallel, it performs the following tasks: (i) controls the monochromator rotation speed and acceleration/deceleration; (ii) uploads and writes a text file with the values of energy and detectors intensity recorded by MUSST for each scan; (iii) corrects for the constant mechanical monochromator backlash-induced shift of the energy scale in the data, between scans carried out in different monochromator rotation directions.

The software has been developed in the spirit of being highly user friendly: the change of scan speed, the scan range and sampling frequency are defined by the software. The output files are multicolumn ASCII files directly usable for the majority of EXAFS analysis software, with a header containing information about acquisition time, scan parameters and the output of different sample environment devices used during the experiment.

Complementary to the implementation of the new acquisition electronics for continuous scanning, we have also developed tools (based on Python and IDL) for the post-processing of the spectra, such as visualization, rebinning, partial integration and fitting, in order to make the handling and pre-processing of the data more efficient.

The ensemble of technological solutions applied is tailored to fulfil the requirement of full inter-operability between continuous and step-by-step scan. An important consequence of this constraint is that we need to maintain the existing monochromator mechanics, and consequently acquisition is limited by two main factors: (i) the maximum monochromator angular speed is 0.42° s^−1^ and (ii) the inversion of monochromator rotation causes a dead-time of ≤1 s. These limitations set a lower limit to our data collection speed. With today’s configuration, a few seconds are required to collect 1900 eV in the range 4–27 keV. The choice of the total acquisition time per spectrum will also be based on the evaluation of the statistical noise level on the spectrum, and therefore in many cases the limitation on the angular speed of the monochromator is not expected to be a major problem.

Fig. 1 ▶ summarizes the new electronics and acquisition chain implemented on BM29: the monochromator lock-in feedback is shown in blue, the step-by-step and continuous-scan acquisition chains in green and red, respectively.

## 3. Test experiments

To illustrate the QEXAFS performance of the beamline we have chosen three classes of materials: (i) standard metal foils, (ii) static data on a catalyst, (iii) one application under working conditions and integration with other techniques. The average signal-to-noise ratio has been evaluated by the ‘chi_noise’ function implemented in the *IFEFFIT* package (Newville, 2001 ▶).

### 3.1. Standard metal foil

**Figure 2:**
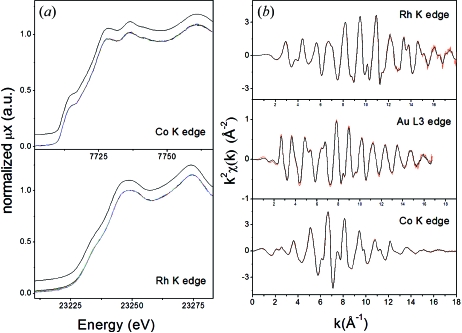
(*a*) XANES spectra of Co and Rh metal foils. The coloured curves are a set of ten QEXAFS spectra randomly chosen on a set of 500 spectra collected in 75 min. The step-by-step scan is shown in black. (*b*) Comparison between EXAFS spectra collected in step-by-step (black curve) and QEXAFS (red curve) modes. The total acquisition time for EXAFS spectra collected in (*b*) is around 4 s.

As already shown in the literature (Ryazhkin *et al.*, 2005 ▶; Stern & Kim, 1981 ▶), one of the main sources of noise in EXAFS spectroscopy derives from sample inhomogeneity. For this reason homogeneous metallic foils are considered the best approximation to the perfect sample. Although the use of metal foils for test experiments tends to hide possible problems of the beamline, we choose to present these results because this is what is mainly presented in the literature and will allow the reader an easy comparison with other facilities. The corresponding results are shown in Fig. 2 ▶. As visible on the XANES (Fig. 2*a* ▶), no apparent loss in energy resolution is detected. It is worth noting that, after software correction for backlash, no difference in edge position is visible between spectra collected when rotating the crystals towards higher or lower angles.

Fig. 2(*b*) ▶ shows a comparison between EXAFS spectra collected by QEXAFS (in red) and step-by-step (black) acquisitions, roughly covering the energy range available today by the use of a Si 111 crystal. Although the QEXAFS spectra have been collected in about 4 s, the difference with respect to spectra acquired in step-by-step mode in 30 min is barely visible.

### 3.2. Catalysts in static conditions

In order to show the performance of the beamline on real samples, different metal-supported catalysts have been measured. These materials have been chosen because they are amongst the most studied by EXAFS spectroscopy, as already predicted by Lytle and collaborators in 1977 at the start of modern X-ray absorption spectroscopy (Sinfelt *et al.*, 1978 ▶).

Metal-supported catalysts have a great industrial interest, being used on a large scale for refining of petroleum, conversion of automobile exhaust, hydrogenation of carbon monoxide, hydrogenation of fats, and many other processes. The metal generally constitutes only a few wt% of the catalytic material, being applied in a finely dispersed form as small particles, often smaller than a few nanometers, on a high-area porous support. The absence of long-range order, the dilution of metal centres, and the need to be characterized *in situ*, coupled with a strong industrial interest, make this class of materials one of the most important for the EXAFS community.

We have chosen to show two commercial catalysts: 5 wt% of Pt on Al_2_O powder from Johnson Matthey, and 0.5 wt% Pd supported on a 1 mm activated carbon grain (TO40) produced by Chimet SpA. In addition, we also tested a homemade 0.5 wt% Pt on Al_2_O_3_ catalyst.

**Figure 3:**
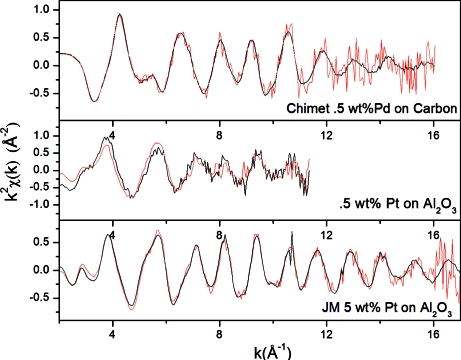
Comparison between EXAFS spectra collected by step-by-step scan (black curve) and QEXAFS (red curve) for the Johnson Matthey 5 wt% Pt on Al_2_O_3_ (5 s spectra), homemade 0.5 wt% Pt on Al_2_O_3_ (40 s spectra) and the Chimet 0.5 wt% Pd on carbon (8 s).

Comparisons between EXAFS spectra acquired using the step-by-step mode and using QEXAFS acquisition are shown in Fig. 3 ▶. We emphasize that the materials used are real samples in the form of pressed pellets, which inevitably leads to an increased inhomogeneity with respect to standard metal foils. The Johnson Matthey catalyst is a good representative of a typical sample of ordinary difficulty (with a total absorption of μ*x* = 2 a.u. and an edge jump around 0.6). For this sample a 5 s scan yields a fully analyzable signal up to *k* = 16 Å^−1^ with a noise-to-signal (N/S) of 8 × 10^−4^. A more difficult sample is the homemade 0.5% Pt on Al_2_O_3_ catalyst, being much more dilute and certainly more suited to fluorescence acquisition rather than transmission, with an edge jump of 0.1 for μ*x* = 2 a.u. In this case a QEXAFS scan obtained in 40 s is of comparable quality to a step-by-step scan obtained in 15 min, with an evaluated noise of N/S ≃ 10^−2^. Finally, the catalyst from Chimet is composed of a grain of carbon of diameter ∼1 mm, with the presence of Pd in low concentration only on the particle surface. On this difficult sample we could obtain an acceptable EXAFS spectrum up to *k* = 12 Å^−1^ within 8 s and a N/S on *k* ^2^χ(*k*) of 3 × 10^−3^.

### 3.3. Hydrothermal synthesis of Ge silicalite

In this last example we perform a hydrothermal synthesis of germanium-doped zeolite. Zeolites are classically defined as microporous crystalline aluminosilicates characterized by the presence of strong acid sites (associated with the Al atoms), and by the uniformity of pore sizes. These characteristics provide these materials with unique properties (high activity and shape selectivity) for their use in heterogeneous catalysis, adsorption and ion exchange operations. The introduction of other elements in the zeolite framework has become more and more popular in recent years. In fact, the introduction of transition metals could also introduce redox functionality, while the substitution of Al could control the acidity of the material. In spite of these advances in the synthesis and applications of zeolites, their mechanism of crystallization is still not well understood.

**Figure 4:**
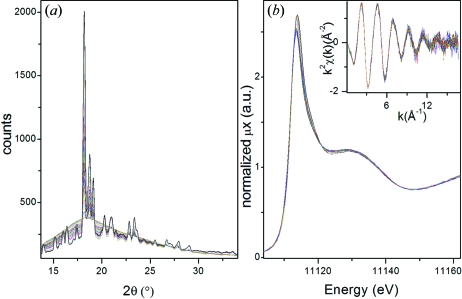
Ge silicalite crystallization process followed by (*a*) powder X-ray diffraction (2 min acquisition) and (*b*) XAS (10 s acquisition).

During this experiment we have followed the crystallization process simultaneously using X-ray diffraction and EXAFS. The availability of both long- and short-range structural information is essential for understanding the kinetics and the role played by Ge on the network formation of the silicalite. Results are shown in Fig. 4 ▶. Diffraction patterns using 2 min acquisition times are shown in Fig. 4(*a*) ▶, and Ge *K*-edge XANES and EXAFS spectra collected between diffraction patterns with a time resolution of 10 s per spectrum are shown in Fig. 4(*b*) ▶.

It is worth noting that, notwithstanding the low percentage of Ge (around 10 wt%) and the presence of almost 2 mm of water on the optical path, the quality of the EXAFS signal is very good with an estimated N/S on *k* ^2^χ(*k*) of ∼10^−3^. Results of this test together with a series of analogue experiments at different temperatures and composition will be discussed elsewhere.

## 4. Future perspectives

In the literature, QEXAFS has shown its utility in the *in situ* observation of several processes. Our implementation on BM29 is characterized by the very high quality of the EXAFS. In the future, we plan to use this quality for other kinds of experiments. One idea is to apply differential EXAFS techniques, successfully developed for dispersive beamlines (Pettifer *et al.*, 2005 ▶), on all samples normally not suited for EDXAS optics. Among these we find samples that modify the energy–direction correlation of the diffracted polychromatic beam (strong small-angle scatterers, for example).

A second idea is to attempt to use QEXAFS acquisition with the achromatic focusing multilayer mirror developed by Ziegler *et al.* (2009 ▶), already tested for the standard step-by-step acquisition. The combination of the two techniques will allow an accurate map to be obtained, where each pixel contains full EXAFS information, on the micrometric scale.

## 5. Conclusion

The implementation of QEXAFS acquisition on BM29 features an excellent signal-to-noise ratio, long time stability and no limitation in the choice of spectrum energy range. The accessible time resolution of a few seconds is sufficient for a wide range of scientific applications and makes it fully complementary to EDXAS or piezo-driven QEXAFS, and to cam-driven monochromator beamlines.

## Supplementary Material

Supplementary material file. DOI: 10.1107/S0909049510046546/hf5176sup1.pdf

## Footnotes

1 Supplementary data for this paper are available from the IUCr electronic archives (Reference: HF5176). Services for accessing these data are described at the back of the journal.
