# Supplementary material for: Quick-EXAFS implementation on the general purpose EXAFS beamline at ESRF
Source: J Synchrotron Radiat. 2011 Jan 18;18(Pt 2):176–82. doi: 10.1107/S0909049510046546 (PMC3267638; doi:10.1107/S0909049510046546)
Supplement: Supplementary file 1 [file s-18-00176-sup1.pdf]

Derivation of formula 5

The I_0 detected signal will be finally multiplied by a digital square wave function $S(\omega t+\phi)$, with the same frequency ω and phase ϕ, and numerically integrated for a time t_i much bigger than the oscillation period (equation 5).

$$Sout(t) = \frac{1}{T}\int_t^{t+T} S(\omega t+\phi) Io(t)dt =$$

$$\frac{1}{T}\int_t^{t+T} S(\omega t+\phi)\left(f(V)+ f'(V)Va\sin(\omega t)\right)dt \approx 1/2 f'(V)Va$$

This formula is based on the property of orthogonality of sinusoidal function, if□ and □ are two intere number then:

$$\frac{1}{\pi}\int_{-\pi}^{\pi} \sin(\mu\vartheta)\sin(\nu\vartheta)d\vartheta = \begin{cases} 0 \text{ if } \mu \neq \nu \\ 1 \text{ if } \mu = \nu \neq 0 \end{cases}$$

$$\frac{1}{\pi}\int_{-\pi}^{\pi} \cos(\mu\vartheta)\cos(\nu\vartheta)d\vartheta = \begin{cases} 0 \text{ if } \mu \neq \nu \\ 1 \text{ if } \mu = \nu \neq 0 \end{cases}$$

$$\frac{1}{\pi}\int_{-\pi}^{\pi} \sin(\mu\vartheta)\cos(\nu\vartheta)d\vartheta = 0$$

This property could be demonstrated by Werner formulas

$$\sin(\mu)\sin(\nu) = 1/2[\cos(\mu-\nu)-\cos(\mu+\nu)]$$
$$\cos(\mu)\cos(\nu) = 1/2[\cos(\mu-\nu)+\cos(\mu+\nu)]$$
$$\sin(\mu)\cos(\nu) = 1/2[\sin(\mu-\nu)+\sin(\mu+\nu)]$$

For first term of orthogonality properties

$$1/\pi\int_{-\pi}^{\pi} \sin(\mu\vartheta)\sin(\nu\vartheta)d\vartheta = 1/2\pi\left(\int_{-\pi}^{\pi}\cos((\mu-\nu)\vartheta)d\vartheta - \int_{-\pi}^{\pi}\cos((\mu+\nu)\vartheta)d\vartheta\right) =$$

If μ=ν:

$$= 1/2\pi\left(\int_{-\pi}^{\pi} d\vartheta - \int_{-\pi}^{\pi}\cos(2\vartheta)d\vartheta\right) = 1/2\pi(2\pi-0)$$

If μ≠ν:

$$= 1/2\pi\left(\int_{-\pi}^{\pi}\cos((\mu-\nu)\vartheta)d\vartheta - \int_{-\pi}^{\pi}\cos((\mu+\nu)\vartheta)d\vartheta\right) = 1/2(0-0)$$

If the integral is performed on a more generic range not a multiple of the period of $\sin(\mu\vartheta)\sin(\nu\vartheta)$ or with ν and μ no more integer the orthogonality properties became approximated.

$$\frac{2}{T}\int_0^T \sin(\mu\vartheta)\sin(\nu\vartheta)dx = \frac{2}{2n\pi + y}\int_0^{2n\pi+y} \sin(\mu\vartheta)\sin(\nu\vartheta)d\vartheta =$$

$$\frac{2}{2n\pi + y}\left(n\int_0^{2\pi} \sin(\mu\vartheta)\sin(\nu\vartheta)d\vartheta + \int_0^{y} \sin(\mu\vartheta)\sin(\nu\vartheta)d\vartheta \right)$$

Where the bigger deviation from the over cited equation is for $\mu = \nu$ and y =π/4μ

$$\frac{2n\pi}{2n\pi + y} + \frac{2\mu y - \sin(2\mu y)}{2\mu y + 4\mu n\pi} = 1 \pm \frac{2}{(8n\mu + 1)\pi}$$

We could generalize for a generic period substituting the variable ϑ with 2p/Lx where L is the period of the function $\sin(2\pi\mu x)\sin(2\pi\upsilon x)$

$$1/\pi\int_{-\pi}^{\pi} \sin(\mu\vartheta)\sin(\upsilon\vartheta)d\vartheta =$$

$$\frac{2}{L}\int_{-L/2}^{L/2} \sin(\frac{2\pi\mu x}{L})\sin(\frac{2\pi\upsilon x}{L})dx = \begin{cases} 0 \text{ if } n \neq m \\ 1 \text{ if } n = m \neq 0 \end{cases}$$

The Fourier series of the square wave function of amplitude π/4:

$$S(\omega t + \phi) = \sum_{n=1,3,5,..}^{\infty} \frac{1}{n}\sin(n\omega x + \phi)$$

The equation 5 became

$$Sout(t) = \frac{1}{T}\int_t^{t+T} \sum_{n=1,3,5,..}^{\infty} \frac{1}{n}\sin(n\omega x + \phi)Io(t)dt$$

in the integral survive only the term with n=1

$$\frac{1}{T}\int_t^{t+T} \sin(n\omega x + \phi)\big(f(V) + f'(V)Va\sin(\omega t)\big)dt =$$

$$1/T\left(\int_t^{t+T} \sin(n\omega x + \phi) f(V)dt + \int_t^{t+T} \sin(n\omega x + \phi) f'(V)Va\sin(\omega t)dt\right) \approx 1/2 f'(V)Va$$
